# Melanocytic or Not? Dermoscopy and Reflectance Confocal Microscopy for Lesions Difficult to Diagnose: A Cross-Sectional Diagnostic Accuracy Study

**DOI:** 10.5826/dpc.1104a127

**Published:** 2021-10-01

**Authors:** Camila Scharf, Giuseppe Argenziano, Gabriella Brancaccio, Gaetano Licata, Andrea Ronchi, Elvira Moscarella

**Affiliations:** 1 Dematology Unit, University of Campania L.Vanvitelli, Naples, Italy; 2 Pathology Unit, University of Campania L.Vanvitelli, Naples, Italy

**Keywords:** reflectance confocal microscopy, dermoscopy, skin cancer, melanocytic, diagnosis

## Abstract

**Background:**

Different techniques for non-invasive skin examination and early diagnosis of skin lesions are available nowadays, being dermoscopy and reflectance confocal microscopy (RCM) the most diffused ones. Several studies supported the complementary use of dermoscopy and RCM that improves diagnostic accuracy when dealing with melanocytic lesions.

**Objectives:**

To analyze RCM diagnostic accuracy in the differential diagnosis between melanocytic and non-melanocytic lesions.

**Methods:**

This is a cohort selected cross-sectional study conducted at the Dermatology Unit of the University of Campania L. Vanvitelli, Naples, Italy, from 2012 to 2020. We searched the image database for all excised lesions for which the clinical and dermatoscopic differential diagnosis was between melanocytic and non-melanocytic and for which an RCM examination was performed. Sensitivity, specificity, and diagnostic accuracy values were estimated.

**Results:**

The study included 53 cases that were found to have disagreement between clinical, histological and RCM diagnosis, of which, in 31 cases the differential diagnosis was melanocytic vs non-melanocytic lesion. The RCM reached a specificity of 87% (95% CI: 0.73–1) and a sensitivity of 62.5% (95% CI: 0.29–0.96) in the present sample. Diagnostic accuracy was 80.6% (95% CI: 0.67–0.94).

**Conclusion:**

RCM has a high specificity in differentiating between difficult-to-diagnose melanocytic and non-melanocytic lesions.

## Introduction

Differentiating between melanocytic and non-melanocytic lesions may be of outmost importance, especially nowadays, when several non-invasive treatment modalities are available for basal cell carcinoma (BCC), solar lentigo (SL) and seborrheic keratosis (SK).

Basal cell carcinomas can resemble scars, intradermal nevi, lichenoid keratosis, seborrheic keratosis, and benign adnexal neoplasms [1]. Squamous cell carcinomas can be difficult to differentiate from hyperplastic actinic keratosis or irritated seborrheic keratosis. Although some melanomas are frequently diagnosed by clinic and dermoscopy, the diagnosis of many melanocytic lesions is undetermined without an analytical approach involving patient assessment, history, pattern analysis, comparison with other patient lesions, and assessment of subtle changes over time.

RCM opened a new era of optical biopsies, with an evident application in the diagnosis of skin cancer due to the high reflective index of melanin and keratin. The mosaic formed during RCM imaging allows a direct correlation between dermoscopy and cytological patterns in the diagnosis of melanoma and non-melanoma skin cancer (NMSC) [2–4].

Recently, studies testing the usefulness of combining RCM with digital dermoscopy monitoring have shown a reduction in the number of lesions excised to diagnose skin cancer, reflecting a 2-fold reduction in unnecessary biopsies [4].

Most of the studies available in the literature discuss the accuracy of RCM in making a diagnosis that corresponds to the histology and/or compare the criteria observed in dermoscopy and/or histology with those observed under RCM examination. However, few studies directly compare the differentiation accuracy between melanocytic and non-melanocytic lesions, the majority focusing on facial lesions [5]. Evaluating lesions on the face was indicated as one of the “best indications” for RCM in a recent study [6].

In this study we aimed to assess RCM diagnostic accuracy in differentiating between melanocytic and non-melanocytic lesions, when compared to dermoscopy and histological examination, the latter being the gold standard for the definitive diagnosis.

## Objectives

To calculate RCM sensitivity and specificity in the differential diagnosis between melanocytic and non-melanocytic lesions.

## Materials and Methods

The study was based on a descriptive data set of consecutive cases for which RCM imaging was integrated in the diagnosis of patients who visited the Dermatology Unit of the University of Campania Luigi Vanvitelli, Naples, Italy, from 2012 to 2020. Patients who attended at the dermatology service between the specified years and had complete data in relation to clinical diagnosis, dermoscopy, confocal microscopy, and histology were included.

The database of the Dermatology Unit includes all images of the excised lesions. Images, RCM identification numbers, preoperative clinico-dermoscopic diagnosis, RCM diagnosis, and the final histologic diagnosis were recorded.

RCM images were obtained using the Vivascope 1500 Reflectant Confocal Imaging System (CaliberID, NY, USA). A minimum of 3 mosaics of 0.5×3×0.5 mm were performed and reconstructed in larger sizes. Composite images were obtained in the granular, spinosum, dermoepidermal junction (DEJ) and papillary dermis layers. RCM examination usually preceedes the sugical excision of about 2 weeks. The definitive diagnoses accepted were histopathologically determined and, in cases where no biopsy was performed, the case was excluded from this study.

Data was recorded in an Excel™ table (Version 14.0.6023.1000, Microsoft Office Professional Plus 2010, © 2010 Microsoft Corporation, Santa Rosa, CA) and submitted to statistical analysis.

Sensitivity, specificity, and accuracy values were estimated considering the histological examination result as the gold standard. In this study sensitivity indicated the probability to diagnose a lesion through RCM, as melanocytic, in accordance with the histology result classifying the lesion as melanocytic. Specificity indicated the probability to diagnose a lesion as non-melanocytic with RCM, with histology results reporting the lesion as non-melanocytic. Accuracy was defined as the RCM success rate in classifying the lesion using the histologic diagnosis result as a gold standard reference. All estimates were calculated on the basis of the studied target population, that is, for cases with a discrepancy between clinical, histological, and RCM diagnosis. Therefore, results cannot be extrapolated and considered valid for all cases in general. The confidence intervals presented for the evaluated parameters are 95%. Were also calculated the likelihood ratio for positive results and the likelihood ratio for negative results. Data was analyzed with Stata/SE v.14.1. StataCorpLP, USA, computer program.

## Results

Search of the database identified 53 cases that presented a discrepancy between the clinical-dermoscopic, histological, and RCM diagnosis. Among these 53 cases, 31 presented diagnostic disagreement between RCM and dermoscopy with respect to the classification as melanocytic or non-melanocytic lesions. Cases of solar lentigo (SL), lichenoid keratosis (LPLK), basal cell carcinoma (BCC), actinic keratosis (AK), seborrheic keratosis (SK), nevi, and melanomas were included. Patients had a minimum age of 9 years and a maximum age of 87 years (mean age: 66 years), being 13 women and 18 men. Regarding the anatomical region, (13) 41% of the cases were in the head/neck region, (9) 25% on the limbs, (6) 22% on the back and (3) 12% other site. The longest time interval between RCM and surgical excision was of 30 days, an acceptable time interval between an index and a reference test.

Over these 31 cases, 23 lesions were defined by histology as non-melanocytic and 8 as melanocytic, and in 86.9% RCM was able to predict the non-melanocytic origin of the lesion, previously classified by the dermoscopy assessment as melanocytic. Among the 8 melanocytic cases, in 5 of them the RCM could indicate the melanocytic origin of the lesion. Sensitivity, Specificity, and Accuracy are shown in Table 1.

In the following table (Table 2), the results of the study are summarized.

## Discussion

RCM revealed a high specificity in defining the melanocytic or non-melanocytic nature of a series of difficult-to-diagnose lesions. On a study sample of 31 lesions (23 non-melanocytic and 8 melanocytic), RCM was able to correctly predict the origin of the lesion in 25 cases (80,6%).

**Figure 1:**
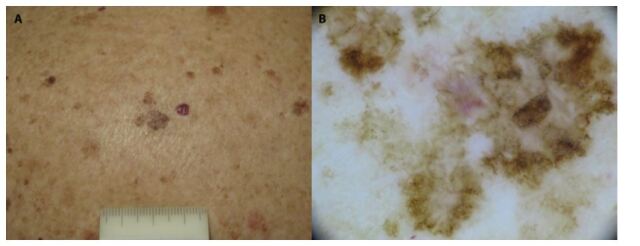
Melanoma in situ on the back of a 75-year old woman. (A) A pigmented macule on a background of intense solar damage. (B) Dermoscopy showing atypical network and regression.

**Figure 2:**
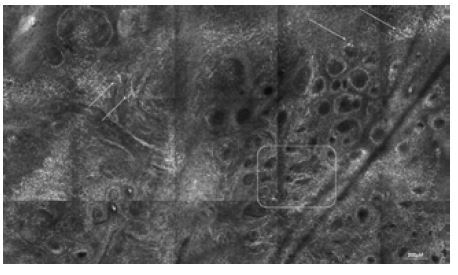
RCM imaging of case 1. Mosaic at the level of the dermal epidermal junction (1.5x2.5 mm), showing meshwork pattern (square). Roundish and dendritic pagetoid cells (arrows).

In previous studies comparing dermoscopy and RCM, we see variable results. Langley et al [7] found no significant difference between the sensitivities (89.2% dermoscopy and 97.3% RCM) or specificities (84.1% dermoscopy, 83% RCM) of the 2 methods. Guitera et al [8] found that RCM had a higher specificity (68%) for the diagnosis of melanoma compared to dermoscopy (68% RCM, 32% dermoscopy), although there was no difference in sensitivity (91% RCM, 88% dermoscopy). However, the differences between specificities were statistically significant, favouring the combination of dermoscopy and RCM over isolated dermoscopy. Finally, Cinotti et al [9] compared dermoscopy and RCM for the diagnosis of lentigo maligna. Unlike previous studies, RCM showed greater sensitivity (80% vs. 61%) and less specificity (81% vs. 92%) when compared to dermoscopy. Thus, the combination of dermoscopy and RCM seems to be the most promising for the diagnosis of melanoma *in situ* [10]. In our study, 2 cases of lesions clinically identified as solar lentigo and 2 as LPLK were later diagnosed as melanoma in histology and in 3 of these cases, the RCM was able to predict the melanocytic origin of the lesion. (Figures 1,2)

Given that conservative treatments are also on the rise, the demand for a reliable approach to non-invasive diagnosis, with a view to a more accurate indication of treatment, is increasing. However, we must consider that the diagnosis of RCM alone, without clinical and dermoscopic information, can lead to overdiagnosis of actinic keratosis and lentigo maligna [11]. In our study, we found 23 cases of lesions diagnosed as melanocytic by dermoscopy, in which RCM was able to predict the diagnosis as being a non melanocytic lesion (solar lentigo and pigmented BCCs in most cases) [11, 12].

**Figure 3:**
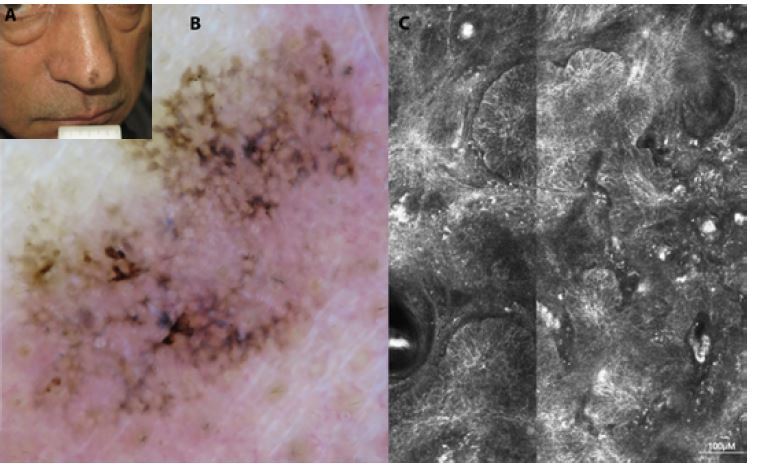
Case 2: Basal cell carcinoma on the tip of the nose in a 60 year-old man. (A) Clinical image: a pigmented macule of 1 cm diameter. (B) In dermoscopy multiple brown concentric structures and peripheral leaf like areas. (C) RCM image showing bright tumor islands.

**Figure 4:**
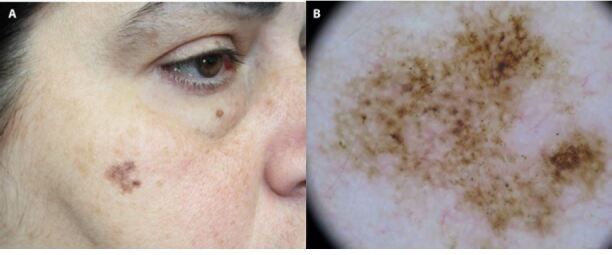
Case 3: Basal cell carcinoma in differential diagnosis with solar lentigo and melanoma. (A) Flat facial lesion on the face of a 40 year-old woman with undefined borders (B) Dermoscopic analysis revealing brown pseudonetwork and grey globules.

**Figure 5:**
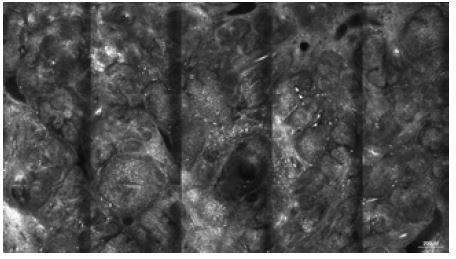
RCM of case 3. RCM Mosaic (2.5 x 1.5 mm) at the level of the upper dermis featuring tumoral islands, typical of BCC.

In turn, when discussing the diagnosis of basal cell carcinoma (BCC), a previous study conducted by Guitera et al [13] analysed 710 consecutive clinically equivocal cases and confirmed that the diagnosis of BCC is relatively accurate with RCM, almost similar to histopathological evaluation (Figures 3–5).

In our study, 2 lesions clinically diagnosed as BCC, later proved to be a melanoma and a melanocytic nevus by both RCM and histology, while out of the 23 lesions clinically thought to be melanocytic, 4 were BCCs, all correctly diagnosed in RCM.

A study by Alarcon et al [14] showed that the use of RCM can decrease the number needed to treat (NNT), when calculating the proportion of equivocal lesions excised for every melanoma. The authors included a set of lesions showing dermoscopic patterns suggestive of melanoma. The analysis of the lesions with dermoscopy alone resulted in an NNT of 3.73, the combination of dermoscopy and RCM resulted in a lower NNT of 2.87, and RCM alone reduced NNT even further to 1.12. There was no significant difference between the specificities of dermoscopy and RCM versus RCM alone.

Another prospective intervention study on a cohort of approximately 1000 patients showed that the number of unnecessary excisions of benign nevi can be reduced by more than 50% using RCM. This reduces the NNE from a potential 14.6 without RCM to a real NNE of 6.8 with the systematic use of RCM in ambiguous lesions [11,15].

The main limitations of our study regard the low precision of the estimated sensitivity and PPV, due to the limited sample size. However, as the RCM is an emerging tecnique availabe only in referral centers, more cases of doubtful melanocytic or not lesions examined by RCM will be available in future.

## Conclusions

RCM showed high accuracy in differentiating between melanocytic and non-melanocytic lesions, especially when associated with dermoscopy.

Although RCM is considered a complementary tool to dermoscopy, it is not clear whether RCM’s diagnostic accuracy depends on the correlation with clinical and dermoscopic information or whether RCM, such as histopathology, functions as an independent procedure. Like most of the studies we analysed, we must consider that diagnosing skin cancer is a very complex process and, whenever possible, we should associate all tools we have at hand, including clinical, dermoscopy, and RCM investigations.

## Figures and Tables

**Table 1:** Statistical Analysis of the RCM When Used to Differentiate Between Difficult-to-Diagnose Melanocytic and Non-Melanocytic Lesions in the Examined Sample.

	Results	CI 95%

**Sensitivity**	62,5%	29,0% – 96,0%

**Specificity**	87,0%	73,2% – 100%

**Accuracy**	80,6%	
**LR+**	+7,81	66,7% – 94,6%
**LR−**	−0,26%	

(LR+)= Likelihood ratio for positive results; (LR−)= Likelihood ratio for negative results

**Table 2:** Results

Histology	RCM	Dermoscopy

8 Melanocytic	1 Melanoma	2 SL
6 Melanoma	1 Spitz Nevi	2 BCC
1 Spitz Nevi	1 SK	2 LPLK
1 Nevus	1 Sebaceous Hyperplasia	2 Dermatofibroma
	1 UNM	
	3 UM	

Non Melanocytic		
3 SL	3 SL	1 Melanoma
2 LPLK	2 LPLK	1 Spitz Nevi
5 AK	2 AK	21 Atypical Melanocytic Lesion
4 BCC	5 BCC	
2 Dermatofibroma	1 Dermatofibroma	
4 SK	1 Melanoma	
2 Vascular Lesion	1 Nevus	
1 Pinkus Fibroepitelioma	1 Pinkus Fibroepitelioma	
	6 UNM	
	1 UM	

SL= Solar Lentigo; BCC= Basal Cell Carcinoma; SK=Seborrheic Keratosis; AK=Actinic Keratosis; UNM= Undetermined non-melanocytic; UM=Undetermined melanocytic; LPLK=Lichen Planus Like Keratosis.
